# High-dose continuous-infusion ifosfamide in advanced well-differentiated/dedifferentiated liposarcoma

**DOI:** 10.1186/2045-3329-4-16

**Published:** 2014-11-22

**Authors:** Roberta Sanfilippo, Rossella Bertulli, Andrea Marrari, Elena Fumagalli, Silvana Pilotti, Carlo Morosi, Antonella Messina, Angelo Paolo Dei Tos, Alessandro Gronchi, Paolo Giovanni Casali

**Affiliations:** Adult Mesenchymal Tumor Medical Oncology Unit, Cancer Medicine Department, Fondazione IRCCS Istituto Nazionale dei Tumori, Via G. Venezian 1, 20133 Milano, Italy; Experimental Molecular Pathology Unit, Department of Pathology, Fondazione IRCCS Istituto Nazionale Tumori, Milan, Italy; Department of Radiology, Fondazione IRCCS Istituto Nazionale Tumori, Milan, Italy; Department of Anatomic Pathology, General Hospital of Treviso, Treviso, Italy; Department of Surgery, Fondazione IRCCS Istituto Nazionale Tumori, Milan, Italy

**Keywords:** Ifosfamide, Liposarcoma, Soft tissue sarcoma, Chemotherapy

## Abstract

**Background:**

Liposarcomas represent the most common histological type of soft-tissue sarcomas (STS). Its main subgroups, WD/DD, is known to be poorly sensitive to chemotherapy, with few active agents, i.e., anthracyclines +/- ifosfamide and trabectedin. High-dose ifosfamide (HDIFX >12 g/m2) is active in STS pts pretreated with standard-dose IFX, though with greater toxicity. A prolonged continuous-infusion (ci) through a portable external pump may be an alternative way to administer HDIFX.

**Methods:**

From March 2002 to August 2013, 28 pts (median age =60, range =37–73 yrs) with advanced disease (6 WD and 22 WD/DD) were given ciHDIFX, at the dose of 14 g/m2 as a 14-day continuous infusion every 4 weeks. Twenty-four pts (86%) were previously treated with chemotherapy (19 with anthracyclines and ifosfamide; 4 with anthracycline monotherapy; 1 with trabectedin).

**Results:**

Seven PR (all in DDLPS), 2 minor response (MR) and 11 SD were observed. Of interest, 6 of 9 patients with PR or MR had had SD with the previous therapy with anthracycline plus ifosfamide. The median progression-free survival was 7 months. Most common side effects were mild myelosuppression (anemia G2-3 in 3 pts; G2-3 neutropenia in 3 pts and G4 in 1; G3 thrombocytopenia in 1 pt); nausea (G3 in 3 pts) and fatigue (G3 in 6 pts). One pts had transient G3 confusion.

**Conclusions:**

These data suggest that ciHDIFX is active in WD/DDLPS, even in patients already treated with a combination of anthracyclines plus ifosfamide. In this series, ciHDIFX regimen was better tolerated than HDIFX in published studies.

## Introduction

Soft tissue sarcomas represent a heterogeneous group of tumours. The most common histological subtype among them is liposarcoma, which comprises three distinct groups: well differentiated/dedifferentiated (WD/DDLPS), myxoid/round cell (MRCL) and pleomorphic. WD/DDLPS is the commonest subgroup and is characterized by distinctive morphology, genetics and natural history [1].

Well-differentiated liposarcoma (WDLPS) are generally indolent tumours and typically affect people between the ages of 40 and 60 years. They occur most frequently in the limbs, followed by retroperitoneum, mediastinum and head and neck region. In the retroperitoneum, WDLPS exhibits a slow but progressive growth over many years without metastasis, but tends to recur frequently and may cause death due to uncontrolled local recurrences, or may dedifferentiate and eventually acquire metastatic potential. The risk of dedifferentiation is related to the site and is probably around 20% in the retroperitoneum but <2% in the limbs [2, 3]. DDLPS may rarely develop as a recurrence of a WDLPS (10% of cases) or may arise de novo typically with a non-lipogenic component that is most often (but not necessarily) high grade [4].

WD/DDLPS have a distinctive genetic signature, as they are characterized by the overexpression/amplification of MDM2, HMGA2 and CDK4 which are currently regarded as a useful diagnostic tool [5, 6].

Treatment for both localized WDLPS and DDLPS is surgery [7], while treatment options for patients with advanced disease are limited. First-line combinations of doxorubicin and ifosfamide in advanced soft tissue sarcoma provide response rates in the 20–40% range [8], with outliers [9]. However, in the subgroup of WD/DDLPS the response rate to anthracycline-based chemotherapy is lower and they are generally considered as a subgroup of poorly chemosensitive soft tissue sarcomas [10, 11]. Trabectedin, approved in the European Union for advanced STS, represents a second-line treatment for WD/DDPLS. However, the objective response rate to this drug does not exceed 10%, if myxoid/round cell liposarcoma is excluded, where the drug is exceedingly active [12]. Recently, in an effort to improve clinical outcome of these sarcoma subtypes, several targeted therapy, such as MDM2 antagonist or CDK4 inhibitors have been studied [13, 14], but results are preliminary and the drug development is ongoing.

Ifosfamide is often used in the second-line chemotherapy setting for advanced sarcoma patients, at high doses, in the range of 12–14 g/m2 over 3–4 days. At these dose levels, ifosfamide may be active also in patients already exposed to standard doses in combination with doxorubicin, though with greater toxicity such myelosuppression, renal toxicity, neurotoxicity, nausea and vomiting [15–17]. Better tolerability using a continuous-infusion regimen were reported [18, 19]. The stability of ifosfamide mixed with mesna within a 7-day pump [20, 21], and recently over a prolonged period of 14 days [22], was assessed.

So, we decided to administer high dose ifosfamide in a prolonged continuous infusion over 14 days (icHDIFX), through two portable external pumps of 7-day duration each. The aim of this retrospective analysis was to review all patients with WD/DDLPS treated with ciHDIFX at our Institution between March 2002 and August 2013.

## Methods

A retrospective analysis of all advanced WD/DDLPS patients treated with ciHDIFX at Istituto Nazionale Tumori of Milan from January 2002 to August 2013 was made. The analysis was approved by the Institutional Ethics Committee. Histological diagnosis was centrally reviewed in all cases by two expert pathologists (SP and APDT). Patients with a WDLP at first diagnosis which, at the time of their treatment with ciHDIFX, had recurred as DDLP as documented at pathologic examination or as suggested by the radiological imaging, are classified as patients with DDLP. In a subgroup of DDLPS, the histological grade of the dedifferentiated component was described according to the FNCLCC grading system. In the subgroup of WDLPS chemotherapy was used only in symptomatic patients, without any chance to be operated. Patients had an Eastern Cooperative Oncology Group (ECOG) performance status (PS) of 0–3, full recovery from toxicity of previous therapy, age >18 years, written informed consent to treatment and data collection for research purposes. Prior nephrectomy was allowed, and patients were excluded only if serum creatinine level exceeded the normal level by 2.5 times. Treatment consisted of ifosfamide 14 g/m2 and mesna equidose, at a dose of 1 g/m2/day for 14 days as follows: ifosfamide (7 g/m2) and mesna (7 g/m2) were mixed 1:1 in normal saline (total volume up to 275 ml) and delivered as a continuous infusion via an external pump over 7 days. The pump had to be replaced after 1 week of therapy, since no data on ifosfamide stability beyond 7 days was available. No hydration was required; oral hydration with 1.5 L/day was recommended. When required, antiemetic prophylaxis consisted of oral ondansetron or granisetron. Full blood count and serum biochemistry were requested at day 1, 8 and 15. No growth factors were included in the treated plan. The chemotherapy cycle was started again on day 28. Treatment was continued until disease progression, unacceptable toxicity, medical decision or patient refusal.

Patient medical records were retrospectively examined to collect clinical data. All patients were evaluated for medical history, physical examination, full blood count and serum biochemistry and a staging computed tomography (CT) or magnetic resonance imaging (MRI) scan. Patients with both local and distant recurrence were classificated as metastatic. Tumor assessment was carried out every two to three cycles. The Response Evaluation Criteria in Solid Tumors (RECIST) was used to assess response [23]. Any radiological reduction in the sum of the longest diameters of target lesions that did not reach the criteria for an objective partial response (PR) was defined as a minor response (MR). The minimum interval to define a stable disease (SD) was considered 8 weeks. Adverse effects were evaluated according to the fourth version of the National Cancer Institute’s common toxicity criteria, based on clinical and laboratory assessments at each cycle.

## Results

### Patient characteristics

Between March 2002 and August 2013, 28 patients with locally advanced or metastatic WD/DDLPS were treated with ciHDIFX on an outpatient basis. Their median age was 60 years (range: 37–73). ECOG PS was 0 in 17 patients (60%), 1 in 5 (18%), 2 in 5 (18%) and 3 in 1. Six patients had WDLPS (5 arising from retroperitoneum, one arising from spermatic cord) and 22 DDLPS (19 arising from retroperitoneum, one from mediastinum and two from spermatic cord; locally advanced = 11; metastatic = 11). In the subgroup of DDLPS patients, the diagnosis was documented at pathologic examination in 20/22 cases and suggested by the radiological imaging in 2/22 cases. In 12 out of the 22 DDLPS patients, we were able to analyze pathologically the dedifferentiated component. This was scored as G3 in 11 cases and G2 in 1 case. Seventeen patients (60%) with retroperitoneal liposarcoma had a prior nephrectomy. Twenty-four (86%) patients were previously treated with chemotherapy: 84% of these patients received conventional-dose ifosfamide (9 gr/m per cycle) associated with anthracycline as their first-line chemotherapy. Four patients (3 DDLPS and 1 WDLP) were treated in first line.

Twelve patients (5 WDLPS and 7 DDLPS) were subsequently treated with Trabectedin, with 2 SD in WDLPS patients previously progressed to ciHDIFX and 2 PR in WDLPS patients who achieved a SD as their best response to ciHDIFX.

The clinical characteristics of patients are summarized in Table 1.

**Table 1:** **Patients characteristics**

Patients characteristics	
Age, years	
Median	60
Range	37–73
PS (ECOG)	
0	17 (60%)
1	5 (18%)
2	5 (18%)
3	1 (4%)
**Gender**	
Male	21 (75%)
Female	7 (25%)
**Histology**	
Well differentiated liposarcoma	6(22%)
Well differentiated/dedifferentiated liposarcoma	22(78%)
**Site of the primary tumour**	
Retroperitoneum	24 (85%)
Spermatic cord	3 (11%)
Mediastinum	1 (4%)
**Number of prior chemotherapy regimens**	
0	4 (14%)
1	21 (75%)
2	3 (11%)
**Prior chemotherapy (24/28 pts)**	
Anthracyclines + Ifosfamide (9 gr/m)	20/24 (84%)
Anthracyclines	3/24 (12%)
Trabectedin	1/24 (4%)

### Drug delivery and toxicity

A total of 105 cycles were administered, with a median of 4 cycles per patient. (range 0–8). The starting dose was reduced to 75% in one patient due to polycystic kidney disease (creatinine value =220 μmol/L with a normal upper value level =105.6 μmol/L). Two patients started treatment with a creatinine value over normal level, without any dose reduction (patients were excluded only if serum creatinine level exceeded 2.5 × normal level) with no documented unexpected toxicities or severe adverse events.

One patient with a PS =3 stopped chemotherapy after two days due to intestinal obstruction related to disease progression. This patient was not evaluated for tumor response.

Seven patients interrupted their treatment in the absence of progression after a median of 7 cycles (range 6–8) as a shared decision with the treating clinician, with a degree of tumour control (PR =4, MR = 1 and SD = 2). One patients interrupted his treatment after 5 cycles with a MR as he underwent surgery of residual disease; other 2 patients withdrew their consent to therapy after two and 4 cycles, following a PR and a SD, respectively.

Two patients interrupted their treatment due to toxicity: one patient had a prolonged G3 thrombocytopenia after one cycle and another had a reversible G3 confusion after 2 cycles.

The haematological toxicities expressed as the lowest value of haematological counts observed during the whole treatment are provided in Table 2. Blood count was normally taken on days 1, 8 and 15 of each cycle. No febrile neutropenia was observed.

**Table 2:** **Haematological toxicity (worst grade per patient)**

Neutropenia	N
G2-G3	3 (10%)
G4	1 (4%)
**Anemia**	**N**
G2-G3	3 (10%)
G4	_
**Thrombocytopenia**	**N**
G2-G3	1(4%)
G4	_

Non-haematological toxicities are reported in Table 3. No renal failure was observed.

**Table 3:** **Non- haematological toxicity (worst grade per patient)**

Nausea/vomiting	N
G2-G3	3 (10%)
G4	_
**Neurotoxicity**	**N**
G2-G3	1 (4%)
G4	_
**Fatigue**	**N**
G2-G3	6 (21%)
G4	_
**Renal failure**	**N**
G2-G3	_
G4	_

### Activity and efficacy

**Figure 1:**
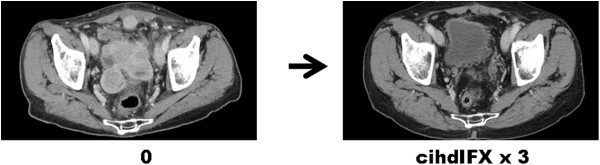
**RECIST partial response in a DDLP.**

Twenty-seven patients were assessed for response. Seven patients (all with DDLPS) achieved a PR by RECIST (26%) (Figure 1), 13 patients (9 DDLP and 4 WDLPS) had SD (48%), with two of them (with a DDPLS) showing minor tumor shrinkage. Overall, tumor control (PR + MR + SD) was >70%. According to histology, PR in the subgroup of DDLPS was 30%.

**Figure 2:**
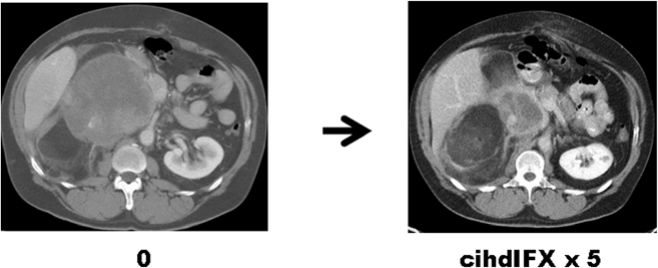
**Dimensional response of the de-differentiated component, while the well differentiated portion stable.**

Radiologically, all observed partial responses seemed to affect the dedifferentiated component. Figure 2 shows a dimensional response of the de-differentiated component, while the well differentiated portion looked stable. All responding patients with DDLP had a G3 dedifferentiated component. One patient with a MR had a DDLP with a G2 dedifferentiated component. All responding patients received ciHDIFX as a second-line therapy, having previously received standard-dose ifosfamide (9 gr/sqm) combined with anthracyclines as their first-line. PR rate in the subgroup of pre-treated patients was 30%.

**Figure 3:**
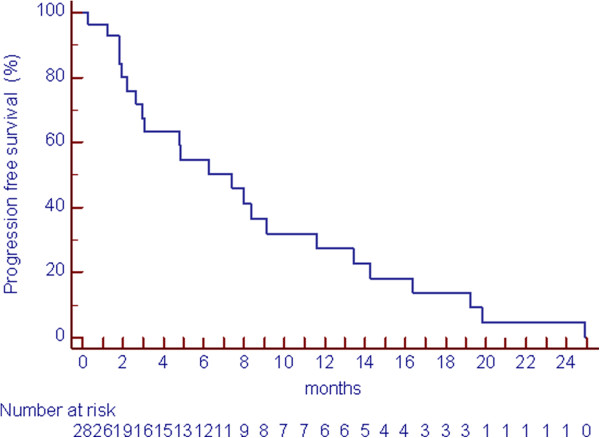
**Progression free survival (PFS).**

The PFS of the whole patients group was 7.4 months. The progression-free rate at 3 and 6 months was 63% and 55%, respectively. Figure 3 shows the progression-free survival curve of all patients.

**Figure 4:**
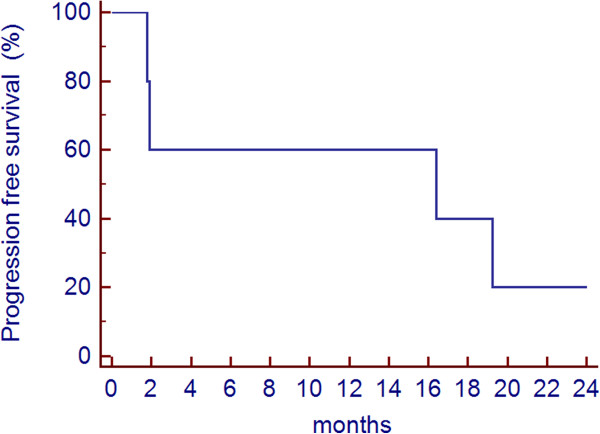
**Progression free survival (PFS) of WDLPS.**

**Figure 5:**
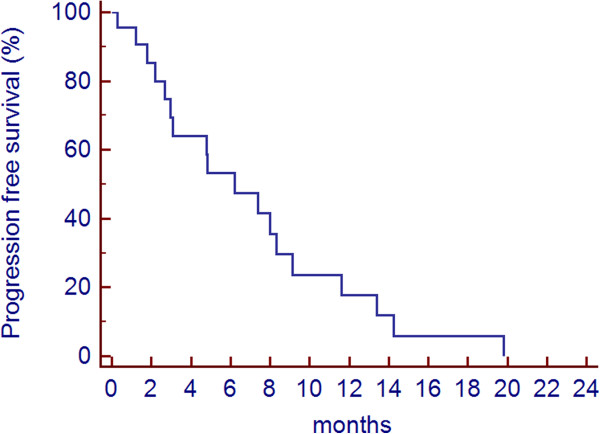
**Progression free survival (PFS) of DDLPS.**

According to histology, the median PFS of DDLPS was 6.2 months (Figure 4), while PFS of WDLPS was 16 months (Figure 5).

## Discussion

In this series of advanced WD/DDLPS patients treated with icHDIFX we observed an objective RECIST response rate of 26% (30% in second/further-line patients and 32% in those with DDLPS), with an additional 50% of patients achieving disease stabilization. Indeed, all PR occurred in patients with DDLPS and all of them had a dedifferentiated high-grade component (Figures 2 and 3). All seven patients with PR and the two with MR had been previously treated with standard-dose ifosfamide (9 gr/sqm) associated with anthracyclines. The median number of cycles was 4, but a subgroup of 10 responding patients interrupted their treatment in the absence of progression after a median of 6 cycles (range 2–8). Of interest, 6 of 9 patients with PR or MR had had SD as their best response with the previous therapy with anthracyclines plus standard-dose ifosfamide, and two patients with SD had had PD as best response to the combination.

Ifosfamide is active in soft tissue sarcomas and is usually included in front-line multi-agent chemotherapy regimens, combined with anthracyclines. Ifosfamide monotherapy is clinically active in advanced adult soft tissue sarcomas: there are many phase 2 studies exploiting different schedules of ifosfamide with a reported response rate between 5 and 39%, with the high response rates achieved using higher doses. Some studies showed the activity of high doses of ifosfamide (≥12 g/sqm) even in patients already treated with standard-dose ifosfamide in combination with anthracyclines. In our series, most patients (75%) progressed on the combination of ifosfamide and antracyclines. Six of 9 patients with PR or MR had a stable disease with their previous therapy. These findings provide further evidence to the capability of HDIFX to circumvent resistance to lower doses of ifosfamide [15–17]. Unfortunately, it is difficult to extrapolate from these studies the response rate in the subgroup of patients with WD/DDLPS and there are no published prospective studies on high-dose ifosfamide selectively focusing on WD/DDLPS.

Indeed, retrospective series reported a low chemosensitivity of WD/DDLPS. In a recent retrospective series of 208 patients with advanced WD/DDLPS, the response rate to chemotherapy with anthracycline-based regimens was reported in 12% of patients, i.e., possibly lower than in other sarcoma subtypes [11]. These results are in line with another report on liposarcoma patients, which showed only a 11% of RR in the subgroup of WD/DDLPS, compared with 44% in myxoid/round cell liposarcomas [10]. Of course it is difficult to compare response rates across different case series, all the more in liposarcoma, given their heterogeneity, being often made up of two components, a well differentiated and a dedifferentiated. In any case, our data on activity of ciHDIFX in WD/DDPLS compare favourably with published evidence. Of interest, in terms of response rate, ciHDIFX looks active in the subgroup of DDLPS, especially with a high-grade dedifferentiation. Also the higher response rate in the subgroup of patients treated in second/further line may be related to the presence of a WDLP between the 4 patients treated in first line. Progression-free survival was higher in the subgroup of WDLPS patients, but, of course, this reflects the slower growth rate associated with this histology. Radiologically, the well differentiated and the dedifferentiated components can be appreciated, since the former has the characteristics of the adipose tissue, while the latter is more dense. However, the recognition of the two components may be flawed by the fact that some well differentiated tumors, i.e. sclerosing and/or inflammatory, may look dense. Figure 2 exemplifies the differential response of an obvious well differentiated component and the dedifferentiated.

The main problem of high-dose ifosfamide given as a 3-day infusion is tolerability, as the studies performed showed myelosuppression, renal toxicity, and neurotoxicity [15, 16, 18] In this sense, a prolonged 14-day continuous infusion of high-dose ifosfamide looks much better tolerated. Though our toxicity data are limited by the availability of blood count and serum biochemistry only at day 1, 8 and 15, no febrile neutropenia was reported and no growth factors were used in our series. No renal failure was reported, even in the subgroup of patients who had prior nephrectomy as part of their treatment for the primary tumour.

Of interest, in the subgroup of 12 patients treated with Trabectedin after progression to ciHDIFX we observed 2 SD and 2 PR in WDLPS patients who had obtained PD and SD, respectively, with ciHDIFX. This suggests the hypothesis that trabectedin might have a different activity profile, being possibly more active in the subgroup of patients with WDLPS who respond less to ifosfamide, and vice versa. If confirmed, this could help personalize treatment choices on the basis of the histology and/or the radiological aspect of the ongoing relapse.

In conclusion, our data suggest that icHDIFX is a valid option for patients with advanced WD/DDLP even if already treated with a combination of anthracyclines and ifosfamide. The activity seems to be higher in those patients with a high-grade dedifferentiated component, and represents a good compromise between activity and tolerability. As this is a small size retrospective series, prospective, possibly controlled, studies are needed to validate these observations. A randomized prospective EORTC study on high-dose ifosfamide versus cabazitaxel will now be set up to confirm these data prospectively.
